# Does biomarker use in oncology improve clinical trial failure risk? A large‐scale analysis

**DOI:** 10.1002/cam4.3732

**Published:** 2021-02-23

**Authors:** Jayson L. Parker, Sebnem S. Kuzulugil, Kirill Pereverzev, Stephen Mac, Gilberto Lopes, Zain Shah, Ashini Weerasinghe, Daniel Rubinger, Adam Falconi, Ayse Bener, Bora Caglayan, Rohan Tangri, Nicholas Mitsakakis

**Affiliations:** ^1^ Department of Biology University of Toronto Mississauga Mississauga ON Canada; ^2^ St Michael's Hospital Toronto ON Canada; ^3^ Institute of Health Policy, Management and Evaluation University of Toronto Mississauga ON Canada; ^4^ Prevention and Cancer Control Cancer Care Ontario Toronto ON Canada; ^5^ University of Miami Miller School of Medicine Coral Gables FL USA; ^6^ Department of Pharmacy Leslie Dan Faculty of Pharmacy University of Toronto Toronto ON Canada; ^7^ Mechanical and Industrial Engineering Ryerson University Toronto ON Canada; ^8^ Institute of Health Policy, Management and Evaluation, and Division of Biostatistics Dalla Lana School of Public Health University of Toronto Toronto ON Canada

**Keywords:** biomarkers, breast cancer, cancer, clinical trial, drug development, lung cancer, melanoma, oncology, risk

## Abstract

**Purpose:**

To date there has not been an extensive analysis of the outcomes of biomarker use in oncology.

**Methods:**

Data were pooled across four indications in oncology drawing upon trial outcomes from www.clinicaltrials.gov: breast cancer, non‐small cell lung cancer (NSCLC), melanoma and colorectal cancer from 1998 to 2017. We compared the likelihood drugs would progress through the stages of clinical trial testing to approval based on biomarker status. This was done with multi‐state Markov models, tools that describe the stochastic process in which subjects move among a finite number of states.

**Results:**

Over 10000 trials were screened, which yielded 745 drugs. The inclusion of biomarker status as a covariate significantly improved the fit of the Markov model in describing the drug trajectories through clinical trial testing stages. Hazard ratios based on the Markov models revealed the likelihood of drug approval with biomarkers having nearly a fivefold increase for all indications combined. A 12, 8 and 7‐fold hazard ratio was observed for breast cancer, melanoma and NSCLC, respectively. Markov models with exploratory biomarkers outperformed Markov models with no biomarkers.

**Conclusion:**

This is the first systematic statistical evidence that biomarkers clearly increase clinical trial success rates in three different indications in oncology. Also, exploratory biomarkers, long before they are properly validated, appear to improve success rates in oncology. This supports early and aggressive adoption of biomarkers in oncology clinical trials.

## Graphical abstract

We reviewed clinical trial success rates in 4 cancers: breast cancer, melanoma, colorectal and lung cancer. We examined the effect of biomarker use on the risk of clinical trial failure. We found that biomarker use best accounted for clinical trial success rates and provides the first extensive statistical analysis to support its use in clinical trial design.

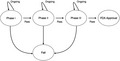

## 1 INTRODUCTION

Cancer continues to be a major challenge in medicine, as it remains the second leading cause of death in the United States, after heart disease,^1^ with a forecasted 1.7 million new cases in 2018.^1^ Finding new treatments remains a challenge, as illustrated by drug failures rates during clinical trial testing for non‐small cell lung cancer (NSCLC; 89%),^2^ metastatic melanoma (83%),^3^ non‐Hodgkin's lymphoma (92%)^4^ and prostate cancer (97%).^5^ In the face of this problem, developing anti‐cancer agents demands new paradigms.

The addition of prognostic and predictive biomarkers, that predict disease progression and a patient's response to therapy,^6^ respectively, have offered promise in tackling the problem of developing new anti‐cancer agents. Biomarker benefits potentially include more cost‐effective use and diminishing the costs of development through better patient selection. Pioneering biomarker approved therapies include: HER2 in breast cancer,^7^ BCR‐ABL in chronic myelogenous lymphoma,^8^ anaplastic lymphoma receptor tyrosine kinase (ALK) rearrangements in NSCLC,^9^ BRAF V600 mutations in melanoma^10^ and the absence of RAS mutations in colorectal cancer.^11^

Published studies to date on the potential benefits of biomarkers in oncology have lacked the analytical rigour, despite pointing to some positive trends.^2^, ^3^, ^4^, ^5^, ^12^, ^13^ In addition, we have argued previously that biomarker use in clinical trial testing can entail new risks.^14^ The current strategy of combining new biomarkers that have never before been validated with a new drug may increase the risk of clinical trial failure. In theory, one would be compounding the probability that a new drug will fail to work with the probability that a new biomarker will fail to target appropriate patients for treatment.

In this study, we conducted the most rigorous analysis of biomarker impact in cancer drug testing in clinical trials published to date. We collected data on clinical trial risk according to the previously published methodology,^2^, ^3^, ^4^, ^5^, ^13^ which was defined as the likelihood that a drug will fail clinical trial testing. Multi‐state Markov models were used to perform the analysis, which involved investigating the association between biomarker use and success rates in the drug progression through clinical trial testing. We used two statistical models, one with biomarker usage as a covariate and the other without, to see which best accounted for the success rates among drugs.

We examined four major cancers with diverse use of biomarkers: (a) unresectable stage III and IV metastatic melanoma; (b) locally or advanced metastatic breast cancer; (c) metastatic stage IIIb–IV NSCLC; (d) metastatic stage IV colorectal cancer. We wanted a clear answer as to whether biomarker use was aiding oncology clinical trial testing for new drugs by quantifying its impact on success rates at each stage of clinical trial testing. To our knowledge, this is the most extensive study of the potential benefits of biomarker use in oncology. Finally, our statistical model was built as an exploratory model to see if we could explain historical data pertaining to the clinical outcomes with biomarkers. The analysis of this study applies to historical data, and more testing is required to turn our model into one that is predictive in future clinical work in oncology.

## 2 METHODS

### 2.1 Drug study and patient eligibility

Detailed descriptions of the methods used for the collection of data and identifying relevant clinical studies in this study have been previously described.^2^, ^3^, ^4^, ^5^, ^13^ Briefly, data regarding trials that pertained to four cancers: unresectable stage III and stage IV metastatic melanoma ('melanoma'); locally or advanced or metastatic breast cancer that failed or had been exposed to anthracycline or taxane chemotherapy ('breast cancer'); metastatic (stage IIIb–IV) non‐small cell lung cancer ('NSCLC') and metastatic (stage IV) colorectal cancer ('colorectal cancer'). Data were obtained from the National Institutes of Health Clinical Trials database (ClinicalTrials.gov) from 1 January 1998 to 2 January 2017.

Trials were included if the investigational drug was a new therapy or a combination therapy (i.e., with standard care) and treated patients with the aforementioned types of cancer. Trials conducted internationally were included if they were registered on ClinicalTrials.gov. Trials were excluded from the analysis if they: (a) initiated their Phase I trial for this indication before 1998; (b) were not industry sponsored; (c) did not treat outcomes related to survival (applicable to Phase II or III trials only); (d) contained trials for topical therapies or new formulations of previously approved drugs; (e) were oestrogen studies in the case of breast cancer. If a drug had been on the market prior to our time interval but only began testing in an indication relevant to us within our time interval, then, such a drug was included in the data set.

### 2.2 Additional databases and online tools

Additional searches using online databases were used to supplement the trial information obtained from ClinicalTrials.gov. The searches included publicly available sites as well as www.archive‐it.org, PR news wire (Factiva), Business Wire (ProQuest 5000) or www.findarticles.com.

### 2.3 Clinical trial outcome classification

**FIGURE 1:**
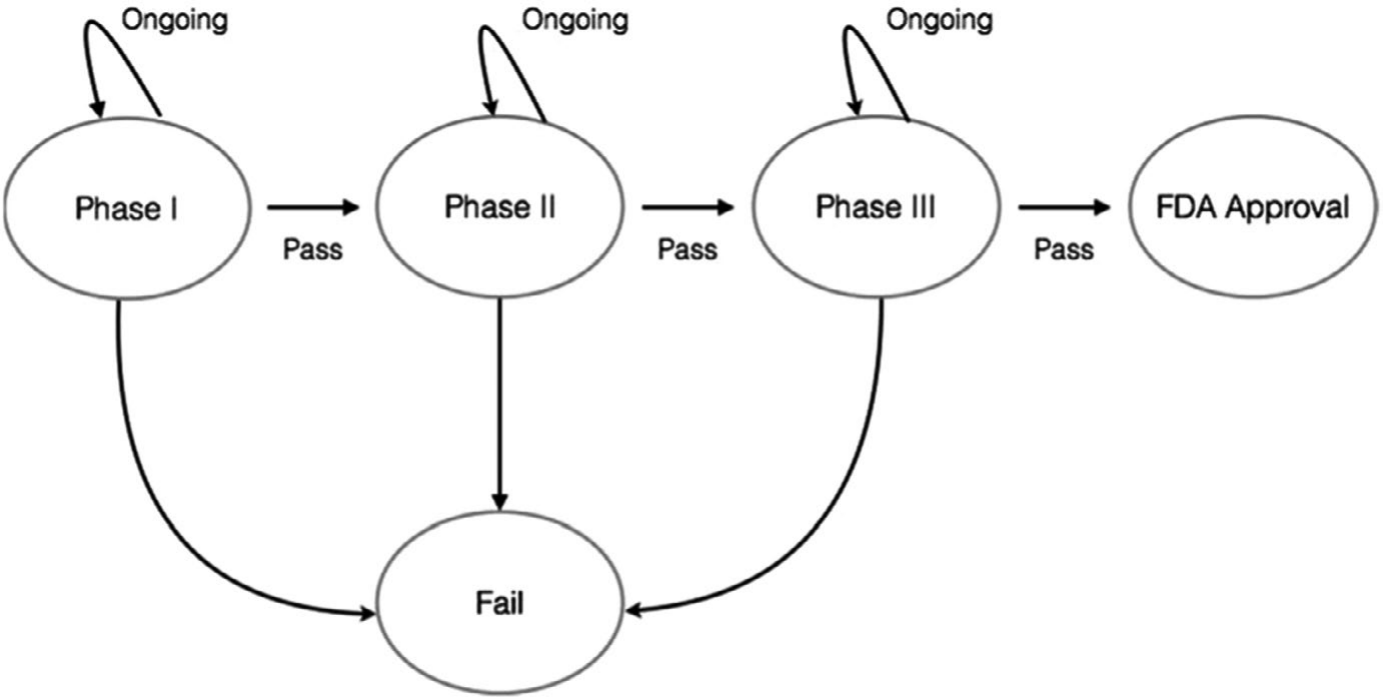
A depiction of five states of clinical trial testing that was used in Markov modelling. Different states used in the Markov model. Biomarker status was a covariate in one model while the other Markov model had no such covariate.

A simple and transparent rule was used to classify the clinical trial outcomes previously described^2^, ^3^, ^4^, ^5^, ^13^ and also depicted here (Figure 1). The phases of development were measured using a standardised definition of success and rested on the following assumptions: a Phase I (or I/II) clinical trial was classified successful if the drug advanced to Phase II for the same indication. Similarly, a Phase II trial was successful if the drug advanced to Phase III clinical testing. Finally, Phase III clinical testing was successful if the drug received approval by the FDA and was available at the time of this analysis. Compounds in active clinical trials were not considered failures or successes. If a treatment had only ongoing trials at a particular phase, that phase was censored from the analysis while previous phases were deemed successful. For example, a drug that has completed phase III but not yet received a decision regarding their submission to the FDA, would be classified as a success for Phase I and Phase II studies, while its phase III status would be classified as ongoing. For a drug to be classified as successfully completing Phase III it must have received FDA approval. Drugs are considered failures: if more than 2 years pass following the completion of a trial and there is no further clinical development; the manufacturer has withdrawn the drug; declared the drug failed to meet its primary endpoints in the study.

### 2.4 Biomarker classification

For the biomarker analysis, only trials that used biomarkers as inclusion or exclusion criteria in clinical trials were included. This analysis focused exclusively on predictive biomarkers. Studies that used biomarkers to confirm the presence of disease were not included in the biomarker arm of analysis. We have defined a biomarker as a biomarker that was used for patient enrolment or exclusion as part of the clinical trial design. We also conducted a sub‐analysis of biomarkers, which segmented this group into two further groups: 'exploratory' and 'validated' biomarkers. Validated biomarkers have previously been approved in the indication in question,^14^ while exploratory biomarkers have not yet had an approval for the indication in question, as part of a drug submission to the FDA. The first time a biomarker is approved by the FDA for a specific indication, it is considered an exploratory biomarker, as are all other biomarkers that are in phase III within 2 years of the approval date. After this 2‐year window, any further use of this biomarker is considered validated and no longer exploratory.

### 2.5 Statistical methods

The primary statistical tool we used in our analysis, multi‐state Markov models, allowed us to address data censoring. Data censoring occurs when the status of a drug is unknown at the time of data collection. Drugs that fail clinical testing or are rejected or approved by the FDA are known outcomes. However, for drugs still undergoing testing in any clinical trial testing phase, it is ultimately not known if the drug will fail in later testing or be approved. Thus, uncertainty in the fate of a drug is a form of data censoring, namely right state censoring in the context of this paper.

Multi‐state Markov models can accommodate censoring in the data. These types of models are used to predict likelihood of a series of events. For instance, in speech recognition used by some smartphones, Markov models––a type of multi‐state Markov model, are used to predict the likely ordering of words in a sentence.^15^ In a multi‐state Markov model, transition intensities measure how quickly subjects are moving between states. In this application, they measure how likely and quickly a clinical trial transition will occur. Clinically, this may mean that drugs that have good data and a positive patient experience, lead to clinical trials that are both more likely to be successful and easier to enrol and meet patient recruitment milestones.

In this study, we used Markov models to see if we can reliably model the sequence of clinical trial transitions (for 'states' see Figure 1). Markov models are used in this study to determine the likelihood a drug in a current clinical trial testing phase will advance to other later phases and subsequent approval. Markov states have been used before in various forms of survival analysis.^15^, ^16^ In this study, we created two Markov models, one with biomarker status as a covariate and the other without. This allowed us to see which model was most reliable in modelling the sequence of clinical trials from one trial to the next. Likelihood ratio tests (LRT) were calculated to compare the fit between models with and without biomarker use as a covariate. In order to express the potential magnitude of our findings, we used hazard analysis (intensity ratios) based on the results from the Markov models. The intensity ratios express how likely clinical trial success based on whether biomarkers were used or not. State censoring is addressed in hazard ratios based on the Markov states. In our sub‐analysis of validated of validated, exploratory and no biomarkers the same approach was used. Markov models were compared as previously conducted, for each of these states. Separate Markov models with different covariates were used: exploratory and validated biomarkers. The ability of Markov models with such covariates to predict trial outcomes was compared to each other and no biomarker Markov models, using likelihood ratio tests as described above.

Multi‐state models, by definition, incorporate transition times (depicted in transition intensities showing the likelihood and the speed of such transitions) and duration of stay in a state as well as transitions from one state to another. A multi‐state model describes how an entity moves between a series of states in continuous time. The likelihood for this model, as used in the R package msm^17^ was calculated from the transition probability matrix. The hazard ratios we report here are the same concept as transition intensities. In a multi‐state model, 'the transition intensities provide the hazards for movement from one state to another'.^18^ Throughout this paper, we refer to this as a hazard ratio rather than its more technical reference known as intensity ratios.

## 3 RESULTS

After employing the search process listed in the methods section, over 10,000 clinical trials were screened, yielding 745 drugs, including the FDA approved drugs, were found that fulfilled our inclusion criteria for all four disease areas. FDA approved drugs in our data set for each indication examined included: breast cancer (11 drugs, 10 with biomarkers), colorectal cancer (6 drugs, 2 with biomarkers), melanoma (6 drugs, 4 with biomarkers) and NSCLC (12 drugs, 7 with biomarkers). Sample sizes in this study refer to the number of drugs, not the number of patients in a clinical study.

An overall analysis of all drugs, irrespective of indication, was conducted based on biomarker status. We compared whether using biomarker status (covariate) in the model more reliably modelled clinical trial transition intensities than a model that did not take into account biomarker status. Independent of clinical trial phase, non‐biomarker transition hazard for drug approval was 2.5 * 10^−4^, confidence interval (CI) (1.36 * 10^−4^, 4.44 * 10^−4^) versus the biomarker model of 1.21 * 10^−3^, CI (0.8 * 10^−3^, 1.8 * 10^−3^). Comparing the Markov models with and without biomarker as a covariate, using likelihood ratio tests, yielded a significant difference (*p* < 2.7 * 10^−10^). While hazard ratios do not address data censoring this is inherent in Markov models using hazard ratios: biomarker hazard ratio, based on the Markov models, was 4.91, CI [2.39, 10.07]. This gave biomarker use, in the context of hazard ratios, a fivefold benefit over non‐biomarker use. Given an overall effect of biomarker as a covariate in reliably modelling clinical trial outcomes, we explored each indication separately by examining the Markov models employing biomarker use as a covariate.

**FIGURE 2:**
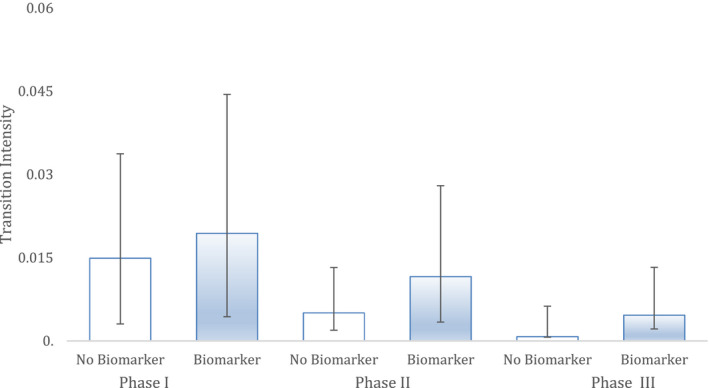
Breast cancer. The ability of two different Markov models to predict clinical trial successes in historical data in this indication. 'Biomarker' shows the performance of a Markov model with biomarker status as a covariate while non‐biomarker has no such covariate. Hazard represents the likelihood and rate of advancing to the next phase. Bars are 95% CI. Sample sizes: Phase I (n = 183), Phase II (n = 132) and Phase III (n = 49).

In breast cancer (Figure 2), the Markov model showed a significant effect of biomarker use in modelling clinical trial outcomes. This was revealed by transition intensities depicted by each phase of clinical trial testing up to approval. When biomarker was used as a covariate it provided superior modelling of outcomes across all clinical trial testing phases. The overall improvement in the fit of the Markov model with biomarker use as a covariate compared to a Markov model with no covariate, compared with likelihood ratio tests, was significant (*p* < 1.73 * 10^−4^). Based on the Markov models, the hazard ratio of drug approval due to the use of biomarkers was dramatic, showing a 12‐fold benefit (HR 12.16; CI [1.56; 94.96]).

**FIGURE 3:**
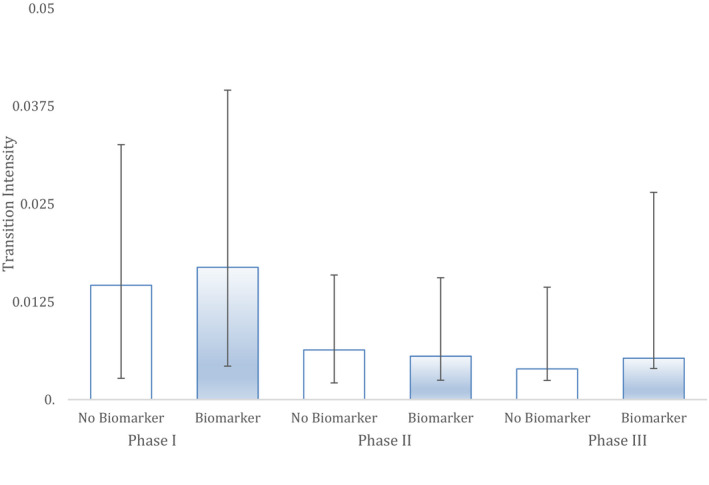
Colorectal. The ability of two different Markov models to predict clinical trial successes in historical data in this indication. 'Biomarker' shows the performance of a Markov model with biomarker status as a covariate while non‐biomarker has no such covariate. Hazard represents the likelihood and rate of advancing to the next phase. Bars are 95% CI. Sample sizes: Phase I (n = 195), Phase II (n = 128) and Phase III (n = 24).

Colorectal cancer analysis did not show a clear benefit of using biomarker status as a covariate in the ability of the Markov model to model future clinical trial outcomes (Figure 3). Trends in transition hazard across clinical testing phases are slight at best. The difference between Markov models with and without biomarker as a covariate, using LRT, was not statistically significant (*p* = 0.85).

**FIGURE 4:**
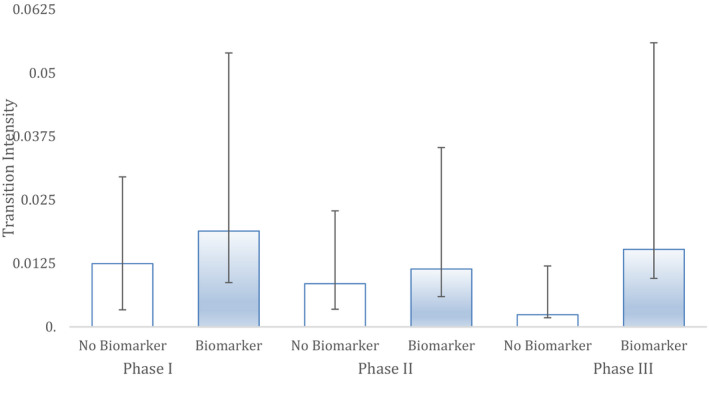
Melanoma. The ability of two different Markov models to predict clinical trial successes in historical data in this indication. 'Biomarker' shows the performance of a Markov model with biomarker status as a covariate while non‐biomarker has no such covariate. Hazard represents the likelihood and rate of advancing to the next phase. Bars are 95% CI. Sample sizes: Phase I (n = 81), Phase II (n = 49) and Phase III (n = 21).

The Markov model with biomarker as a covariate modelled melanoma clinical trial success more reliably than a model with no such covariate, across all clinical trial testing phases (Figure 4). The melanoma Markov model that incorporated biomarker use as a covariate compared to the model with no such covariate was different at a statistically significant level (*p* < 0.029). Hazard analysis based on the Markov models showed the greatest benefit of biomarkers to be in the transition from Phase III to approval (HR 6.36; CI [1.16, 34.71]). The overall impact of biomarker status on drug approval, based on hazard analysis from the Markov models, showed an eightfold impact (HR 7.98; CI [1.46; 43.56]).

**FIGURE 5:**
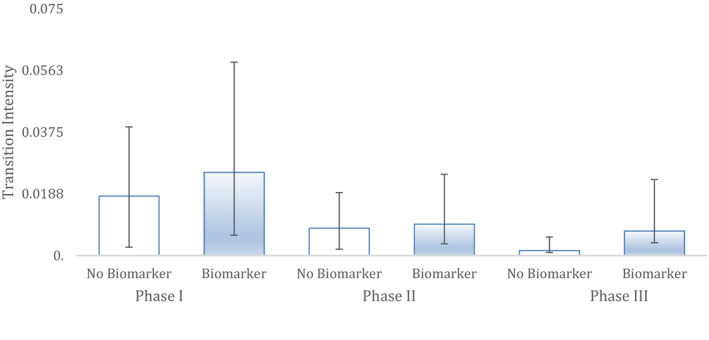
NSCLC. The ability of two different Markov models to predict clinical trial successes in historical data in this indication. 'Biomarker' shows the performance of a Markov model with biomarker status as a covariate while non‐biomarker has no such covariate. Hazard represents the likelihood and rate of advancing to the next phase. Bars are 95% CI. Sample sizes: Phase I (n = 286), Phase II (n = 217) and Phase III (n = 65).

Biomarker status appeared to have an effect in NSCLC cancer (Figure 5). The NSCLC Markov model with biomarker status as a covariate was statistically significant compared to the Markov model without such a covariate (*p* < 2.6 * 10^−4^). This effect was seen in the transition intensities for Phase I to Phase II and Phase II to Phase III. Overall, the hazard ratio from the Markov model showed a clear benefit of biomarkers on the likelihood of drug approval (HR 6.89; CI [2.02, 23.54]).

**FIGURE 6:**
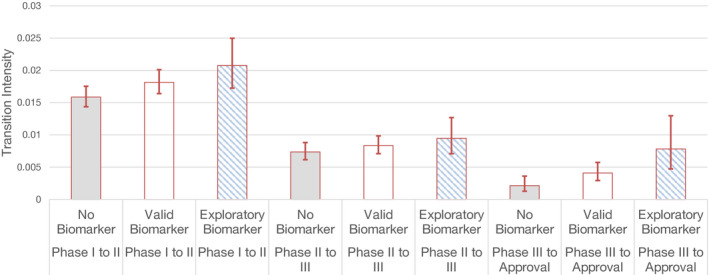
A sub‐analysis of biomarkers into exploratory and validated biomarkers, respectively. The performance of Markov models using each of these biomarkers as covariates is depicted alongside Markov models that do not look at biomarkers. Clinical studies with exploratory biomarkers outperform clinical trials with no biomarkers. Total study sample size of 748 drugs was segmented into: no biomarker (n = 555 drugs), validated biomarker (n = 80) and exploratory biomarker‐based drugs (n = 113). Bars are 95% CI.

Finally, we conducted a sub‐analysis of the biomarkers by collapsing all indications into a single group (Figure 6). Biomarkers were divided into validated and exploratory biomarkers. The biomarkers found in this study (Table 1) include both validated and exploratory biomarkers for the indications reported in this paper (Table 2). First, there was no difference between Markov models that used exploratory biomarker covariates versus validated biomarker covariates, in their ability to reliably model the data (*p* > 0.05). If we compare a Markov model with a covariate for biomarkers (irrespective of the type), in this analysis which has combined all indications, it does better reliably modelling the clinical trial outcomes than a Markov model without a covariate for biomarkers (*p* < 1.3 * 10^−6^). Importantly, Markov models that used exploratory biomarkers as a covariate modelled clinical trial success rates across all clinical trial phases more reliably than a Markov model with no biomarker covariate (*p* < 8.0 * 10^−5^). Overall, the hazard ratio comparison between exploratory biomarkers and no biomarkers was 4.6 (CI 2.0458298, 10.5073327).

**TABLE 1:** Biomarkers that were identified in the four cancer indications and included in this study. Biomarkers were set into categories based on the level of validation for the cancer in question. Biomarkers were either approved for the cancer of interest, are under investigation but have been approved in for use in another indication or have no approval history whatsoever ('exploratory'). A biomarker can be classified as exploratory upon initial approval, and any clinical studies involving that biomarker in phase III within 2 years of the approval date. However, after 2 years of the FDA approval date for the biomarker, any additional work with this biomarker was classified as 'validated'

Disease Area	FDA Approved in this Indication	FDA Approved in Other Disease Areas	Exploratory
Colorectal Cancer	KRAS (Kirsten Rat Sarcoma) gene	BRAF V600 mutation	Beclin 1, p62 and LC3 biomarker
		UGT1A1*28 allele	
			RAS
Breast Cancer	HER2 overexpression		BRCA1 & BRCA2
	Hormonal positive		
NSCLC	EML4‐ALK gene	KRAS gene	MEK1 gene
	EGFR	PD‐L1	N‐ras mutation
	ALK	BRAF V600E	HLA‐A haplotype
		HER2	P13 K activation
			TrkA, LFT3, VEGFR, PDGFR, FGFR
			MET
			FGFRI
			IGF
			ROS1
Melanoma	BRAF V600 mutation	C‐kit (D816 V mutation negative)	SPARC MAGE–A3 s
			CDK (Cyclin D−1)
			HLA‐A haplotype 0201
			ICAM−1 & DAF
			Aurora kinase
			CDK4/6
			N‐cadherin
			DDR2
			MEK
			N‐ras mutation

**TABLE 2:** A summary of biomarker and non‐biomarker drugs by indication in the study. A supplementary file of the data has been submitted to the journal

Indication	Biomarker	No Biomarker	Grand Total
Breast	70	113	183
Colorectal	61	134	195
Melanoma	10	71	81
NSCLC	240	46	286
Grand Total	381	364	745

## 4 DISCUSSION

Drug development in oncology continues to see high failure rates, with several studies estimating almost 90% failure rate of all drugs entering clinical trial testing.^2^, ^4^, ^5^ Biomarkers have been successfully applied in a number of conditions, but if they are not fully validated, may add to the risk of unsuccessful clinical trial outcomes.^14^ This is the first study to conduct a rigorous quantitative systematic large‐scale analysis of the impact of biomarkers on clinical trial risk in oncology. We used two Markov state models, where one incorporated biomarker use status as a covariate, and compared how reliably each model sequenced clinical trial success. Four cancers were examined based on their use of biomarkers. Our overall analysis of these four cancers, independent of indication, revealed a fivefold benefit of hazard ratios from the Markov models, suggesting a substantial benefit from biomarker use. The hazard ratio analysis of the Markov biomarker models examined how likely clinical trial success was associated with biomarker use versus no biomarker use. Hazard ratios indicated that for biomarker‐based drugs clinical trial success was largest for breast cancer (12‐fold) followed by melanoma (eightfold) and lung cancer (sevenfold). While colorectal cancer showed no overall benefit of biomarker use, this finding may change with future clinical work in this area. Finally, Markov models with exploratory biomarkers outperformed (fourfold) Markov models with no biomarkers. Our data provide the most extensive look at biomarker use to date in oncology, with an advanced statistical method. Our findings indicate that biomarkers provide a statistically significant benefit, despite the fact our study includes biomarkers not yet FDA approved.

Biomarker use in clinical trials may be, in itself, predictive of a success in some indications in looking at our historical data. For example, the 12‐fold hazard ratio we observed in our data set for breast cancer from the Markov states indicates that clinical trial success is 12 times more likely in biomarker‐based clinical studies than in studies without a biomarker. This finding suggests that clinical trials involving biomarker use in breast cancer should be given a priority for patient participation compared to trials in this indication that do not use a biomarker. Other indications examined here also showed a powerful effect of biomarker use. NSCLC had a sevenfold increase in hazard ratio predicting clinical trial success in our data, compared to a model with no biomarker covariate. There are of course biomarker approved drugs for NSCLC (crizotinib^19^ and gefitinib^20^), but the results here suggest that such success is not specific to the choice of biomarker, rather, a broader benefit is conferred by biomarkers as a tool themselves. This is surprising, since most of the biomarkers reported in this paper (Table 1) are not validated through an FDA approval for the indication in question. It would be different, for example, if the success of melanoma were driven by a single biomarker that was FDA approved. Quite the contrary, melanoma has the largest preponderance of exploratory stage biomarkers compared to the other indications reported in this study. This suggests that while biomarkers that have not been fully validated may bring additional risk that a clinical trial will fail,^14^ there may be enough benefit to their use to offset the risk of the unknown.

Testing both a drug and a biomarker does introduce two sources of risk to a study and this has been reviewed previously.^21^ It has been argued that if type I (false positives) and type II (false negatives) errors are optimised in a proof of concept study, each can be tested simultaneously in the same study while having feasible requirements for patient enrolment. While misgivings had been expressed before about testing new biomarkers and drugs simultaneously in terms of aggregate clinical trial risk,^14^ increasingly this looks like a practical approach. An extensive model has been developed that lays out a strategy for co‐developing a new drug and new biomarkers as part of a single clinical trial programme.^21^ This approach, in essence, constantly updates the development of a novel biomarker with a novel drug by incorporating maturing phase II data into the phase III study, up until the point of an interim analysis ('phase II+'). This strategy also posits multiple small proof of concept clinical studies with an exploratory biomarker rather than a single larger early clinical study in order to explore more hypothesis about biomarkers for the drug. The results from our exploratory biomarker analysis in this study support this approach. Exploratory biomarkers, given the current conditions in which they are developed and screened with animal data, appear to be much more predictive than investigators would have anticipated. In this model, the level of certainly around exploratory biomarkers has impact on the choices available to the development team on how to proceed. The results reported here suggest that optimism for exploratory biomarkers can be relatively high, and in that context, may upward adjust probabilities of biomarker success derived from Phase II data ('posterior probabilities'). Finally, the model also lays out a biomarker stratified Phase II design where biomarker‐positive and biomarker‐negative exist with both control and treatment arms. The authors note that this the best approach where there is uncertainty the ability of the biomarker to identify likely responders (or non‐responders) for the drug. We concur with the model's conclusion that co‐development of a novel drug and an exploratory biomarker, may indeed be more cost‐effective and faster based on our analysis.

This research has a number of limitations, some of which have been discussed in the literature before.^3^ Phase I cancer studies tend to include patients with different kinds of cancers, even though the drug will eventually be tested in one specific indication in Phase II and Phase III trials. In this sense, because Phase I oncology trials do not usually enrol a single type of cancer patient, they truly reflect oncology more broadly rather than a specific indication. In addition, the indications we are working with are broad categories that contain patient subgroups that may have different molecular subtypes of the disease. For these molecular subtypes of the indication, they may be different with respect to the utility of biomarkers and the course of their disease. As the number of clinical studies grows, it may be possible to analyse these subgroups of patients in the future. With respect to our sub‐analysis of biomarker types there is a concern that our sample size is too small to support this analysis in this data set. There are assumptions in that analysis that need to be explored further, such has lengthening or shortening the period after a biomarker is approved where subsequent use of the biomarker is no longer considered exploratory.

For oncologists enrolling patients in clinical trials our study suggests that with the possible exception of colorectal cancer, biomarker‐based studies should receive priority. More specifically, oncologists may take a more favourable view towards exploratory biomarkers in reducing clinical trial risk.

## CONFLICT OF INTEREST

Parker has worked in the pharmaceutical industry. Dr. Lopes has received honoraria, has had a consultant role and has received research funding from Pfizer, Merck Serono, Roche, AstraZeneca and Eli Lilly in the past.

## Supporting information

Supplementary Material

Supplementary Material

## Data Availability

Publicly available upon request. Data were pooled across four indications in oncology drawing upon trial outcomes from www.clinicaltrials.gov: breast cancer, non‐small cell lung cancer (NSCLC), melanoma and colorectal cancer from 1998 to 2017.
